# Treatment of Tuberculous Glands

**Published:** 1911-01

**Authors:** Emory Lanphear

**Affiliations:** Professor of Surgical Anatomy and Clinical Surgery in the American Medical College, St. Louis, Mo.


For Texas Medical Journal.

### Treatment of Tuberculous Glands.

BY EMORY LANPHEAR, M. D., PH. D., LL. D., PROFESSOR OF SURGICAL ANATOMY AND CLINICAL SURGERY IN THE AMERICAN MEDICAL COLLEGE, ST. LOUIS, MO.

Tuberculous glands which show a decided tendency to grow, or rapidly increase in number, unquestionably should be removed and vigorous anti-tubercular treatment instituted: out-door life, forced feeding, attention to excretions, etc.

But when the glands are quiescent they should be left alone, as a rule, unless disfiguring—as on the neck. However, they sometimes may be made to disappear under the local use of the official iodine ointment (unguentum iodi, U. S. P.). When ordered for children with very delicate skin this should be diluted with an equal quantity of lanolin. It should be applied with gentle friction twice daily. This method of applying iodine is preferable to painting with tincture of iodine—the treatment empirically used by the last generation for "scrofulous" glands. The treatment must be discontinued as soon as it is evident that liquefaction is taking place.

This liquefaction is not true abscess-formation; it is the disintegration of the caseous product of bacterial action—the bacillus tuberculosis—and the material will show, on microscopic examination, none of the true pyogenic micro-organisms. For this reason, if excision is attempted, the closest attention must be paid to secure absolute asepsis; and also not to break the envelop during enucleation, since if it be opened tubercular matter may be forced into the tissues, with disastrous results. In some cases, hematogenous infection with pus-germs may occur and inflammation (adenitis) follow—in which cases alone are incision and curettage permissible.

Over the proper treatment of glands beginning to break down ("cold abscesses" or "scrofulous abscesses" of the older writers) there has been much dissension.

In preantiseptic days surgeons generally refused to operate on account of what we now call mixed infection: introduction of pyogenic micro-organisms with consequent long-continued suppuration, great deformity or even death from chronic asepsis in bad cases.

With the perfection of the aseptic technic practically all operators adopted the plan of excision (without rupturing the envelope of the "abscess") and closure without drainage, hoping to secure primary union, without much scar on exposed surfaces. But in many cases immediate union was not secured, secondary infection with pyogenic micro-organisms occurred and results were unsatisfactory. Hence, many surgeons have fallen back on aseptic removal of the contents of the gland through a canula (by forcible expression after removal of the trocar) and the injection of some antiseptic solution—the majority favoring iodoform or iodine on account of its special antagonism to the tubercle bacillus.

In France the non-operative treatment has gone so far that not even aspiration is practiced—patients are, when possible, sent to the country or seashore and made to sleep in the open, just as in pulmonary tuberculosis, give large quantities of highly nutritious food, with fixation of the affected region and rest in bed if any elevation of temperature occurs; and injection of small doses of tuberculin: third decimal dilution. Under this plan of treatment many cases recover, but most have to be subjected to either excision or aspiration.

The point of puncture should be as high as possible to prevent formation of a fistula. Simple withdrawal may sometimes be sufficient, though in most instances repetition of the aspiration is necessary. If the cavity refill again and again, some injection will be required. Murphy advises use of 2 per cent formalin in glycerine; which must be prepared at least twenty-four hours before injection. Most surgeons prefer a 5 per cent emulsion of iodoform and glycerine, freshly sterilized.

An improvement on this is claimed by Calve and Gauvain (*London Lancet,* March 5, 1910) in this formula:

| | |
|---|---|
| Iodoform | 5 |
| Ether | 10 |
| Guaiacol | 2 |
| Creosote | 2 |
| Olive oil | 100 |

Immediately after injection, the heat of the body volatilizes the ether and deposits the iodoform, as a fine powder, upon the walls of the abscess-cavity. Ten to 20 c.c. (two drams to half an ounce) of the solution, vigorously shaken before using, may be injected.

This line of treatment gives a favorable result in fully 95 per cent of cases treated before the "abscess" is near rupturing; in which cases (about 5 per cent) sinuses form.

Unfortunately, many patients do not come to the doctor until rupture has occurred, with mixed infection and sinus-formation. Here, again, opinions of surgeons differ as to proper procedure. If there is but one gland affected, it is best to remove it (generally under local anesthesia) and allow the wound to heal by granulation from the bottom. If numerous other glands are enlarged but not suppurating the abscess may be dissected out and the other glands permitted to remain until perfect healing is secured; then excised or otherwise treated as seems best.

When granulation is slow it may be stimulated by use of balsam of Peru; or by the application of scarlet red.

Cases not yielding to the iodine locally, tuberculin hypodermically (once or twice a week—not oftener, and in small dosage only), out-door life, good food, etc., may be treated with the X-ray if operation is denied or regarded as inadvisable. X-ray treatment requires about three months, at the end of which time —if favorable results have been obtained—the glands have become shrunken and hard: mere fibrous nodules which, as a rule, never give any further trouble. Radiation must be intense, with tube placed about twelve inches from the surface in order to influence the whole of the diseased area. The secret of success is to have the tube placed at proper distance, giving off rays which are rich chemically and of great penetrating power. Greatest care must be exercised, therefore, not to produce X-ray burns; and the remainder of the body must be scrupulously protected against baneful effects of this powerful, and much abused, agent.

In every case of enlarged glands of the neck the tonsils should be inspected and, if presenting even slight evidences of disease, should be removed.

---

We are permitted by the author to publish the following beautiful lines. It has been set to music and published by the B. F. Haviland Music Publishing Company, of New York. It can be had at the music stores. Price, 25 cents. Colonel Walter is the Representative from Gonzales in the Thirty-first Legislature.

"IN HASTE THE LOVING MESSAGE BEAR."

Words by Chas. K. Walter, Gonzales, Texas.

In haste the loving message bear,
To one who is far away;
And say for us, when thou art there,
Though long has been her stay;
We, in our hearts, her image wear,
We think of her each day.
We pray that heav-en-ly care
Will guard her lonely way.

CHORUS.

May the good an-gel al-ways stand
By ma-ma's and pa-pa's girl.
Leading her ever by the hand
Through this mer-ci-less world.

She must come back to her dear home,
Where moth-er will ca-ress,
Father longs for her to come,
O, could he ask for less?
She's not the first dear one to stray
To de-vi-ate from right,
And our loved one can not stay;
She's coming home tonight.

